# Effective Behavioral Changes through a Digital mHealth App: Exploring the Impact of Hedonic Well-Being, Psychological Empowerment and Inspiration

**DOI:** 10.2196/10024

**Published:** 2018-06-15

**Authors:** Yuting Lin, Carina Tudor-Sfetea, Sarim Siddiqui, Yusuf Sherwani, Maroof Ahmed, Andreas B Eisingerich

**Affiliations:** ^1^ Imperial College Business School Imperial College London London United Kingdom; ^2^ Digital Therapeutics, Inc London United Kingdom

**Keywords:** mHealth, gamification, cognitive behavioral therapy, empowerment, well-being, inspiration, mobile app, behavior change and prevention, digital

## Abstract

**Background:**

New mobile health (mHealth) software apps are emerging and are providing the foundation to radically transform the practice and reach of medical research and care. For this study we collaborated with Quit Genius, a cognitive behavioral therapy (CBT) based mHealth app that helps users quit smoking, to explore the effective design of a digital mHealth app; one that delivers important benefits to its users and helps them change their behaviors for a healthier lifestyle.

**Objective:**

The specific aims of this study were to (1) explore the key role of CBT program progress, (2) examine the gamification design app elements that deliver significant benefits (ie, empowerment, well-being, inspiration) to users, (3) explore the effectiveness of these app elements to help users quit smoking or reduce the number of cigarettes smoked, and (4) identify and describe any potential drivers and hindrances arising from the app design elements.

**Methods:**

We developed an online survey and sent an email invitation to 4144 individuals, who had previously or were at the time using the Quit Genius mHealth app, to encourage participation in the study. We matched the online survey data with objective app usage data of the study participants.

**Results:**

A dataset of 190 completed responses was used. At the time of the survey, respondents had completed an average of 60% of the CBT program in the Quit Genius mHealth app. Of the respondents, 36.3% (69/190) noted to have quit smoking successfully after using the Quit Genius app. As for those who remained smokers after using the app (N=121), the number of cigarettes smoked per day was significantly reduced by 59.6%. The ability of the app to enhance users’ hedonic well-being and psychologically empower them in their daily lives was identified as being key in helping users quit smoking. Specifically, the results show that users whose well-being was enhanced through the app were 1.72 times more likely to quit smoking successfully. Moreover, a one-unit increase on a 7-point Likert scale in the app’s ability to empower smokers in their daily lives led to a reduction of cigarettes smoked per day of 53%. The app’s inspiration to users, however, was negatively associated with quitting success and the reduction in cigarette smoked per day.

**Conclusions:**

The findings offer important insights for the effective design of digital mHealth apps. Specifically, we find that perceived psychological empowerment and enhanced hedonic well-being from the mobile solution may be a more impactful way to support the effectiveness of mobile cognitive behavioral therapy for smoking cessation than eliciting strong inspiration.

## Introduction

These days, many people look at their phone hundreds of times per day [1] and it is digital services such as Instagram or WeChat that extensively permeate peoples’ lives [2-4] and dramatically affect their personal well-being, either positively, by connecting people, or negatively, by creating stress and anxiety [5]. This very high engagement level with one’s smartphone offers an opportunity for mobile health (mHealth) apps to help people lead healthier lifestyles and engage in positive health behaviors such as quitting smoking.

Gamification, defined as “the use of game design elements in non-game contexts” [6], can serve as a natural bridge between the existing innate nature of play, and repurposing games to enhance people’s health. Tailoring motivational affordances or “gamification tactics” to a task has been noted to be critical to successful gamification [7,8]. Goal-setting, for instance, has been noted as an effective tool for enhancing self-motivation [9]. Moreover, enhancing self-motivation incites the “wanting to do it oneself” psychology that is present from childhood and integral to an individual’s concept of self [10]. It is worth noting that individuals proactively seek play, something that gamified experiences theoretically automate [11,12]. With the advent of digital multimedia and resulting virtual worlds, there has been a surge in multidisciplinary interest in the short-term and longer-term applications of gamification [13,14].

Gamification revolves around a complex interaction between physical (eg, vision or motion), psychological and social domains. These domains can be understood to be driven by an intrinsic motivation to satisfy human needs [15]. Gamification elements act as affordances to enhance intrinsic motivation, leading to different psychological states such as arousal, excitement, and contentment [15,16]. In turn, these may drive behavior change. Gamification is increasingly used as a design strategy when developing behavior change support systems in the healthcare domain [17]. For instance, studies have shown that aspects of gamification and program progress in gamified applications can be twice as effective in frequency of self-monitored exercise for weight loss programs compared to a standard paper diary [17]. Ultimately, applications that incorporate behavior change techniques and program progress thereof tend to be associated with increased intervention effectiveness [18,19].

In this study, we examined how various gamification tactics affect behavior change by psychologically empowering users, enhancing their well-being, and giving them inspiration. We identified challenge–ability balance, meaningful framing, personalization, fun or user-centric design as key gamification tactics for our study based on prior research [16,18]. Such gamification tactics, among others, have been identified to elicit behavioral change within the health and healthcare settings, yet there is no clear pattern as to the type of behavioral change strategy that is most effective [8,18].

We complement and extend prior work by testing to what extent, if at all, mobile health (mHealth) solutions can positively impact an individual’s well-being [20]. In our study, well-being is defined as the ability of the mHealth app to contribute to users’ happiness, increase their overall life satisfaction, and help them become more productive at work [21,22].

Specifically, building on and extending prior research that studied behavioral change in the context of gamification [18,23], our research asks whether inspiration (the extent to which the mHealth app, through a gamified journey, motivates users to be the person they want to be, and inspires users to live a healthier life) can be achieved through gamified app design and explores its impact on behavioral change. Based on prior work on psychological empowerment, empowerment is defined as intrinsic motivation that manifests in four cognitions; meaning, competence, self-determination, and impact [24,25]. In the context of our study, we tested the extent to which the gamification design of an mHealth application helps users think of behavioral change differently, realize that mobile apps can improve their health, increase willpower and ultimately change their behavior.

**Figure 1:**
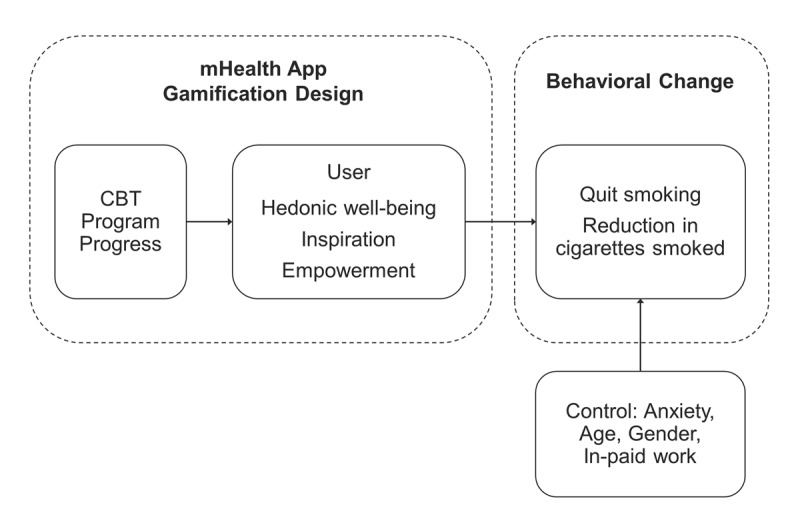
Research model. CBT: cognitive behavioral therapy; mHealth: mobile health.

For this research, we conducted an online survey with users of a cognitive behavioral therapy (CBT)-based mHealth app that helps them quit smoking to (1) explore the key role of CBT program progress, (2) examine the gamification design app elements that deliver significant benefits (ie, empowerment, well-being, inspiration) to users, (3) explore the effectiveness of these app elements to help users quit smoking or reduce the number of cigarettes smoked, and (4) identify and describe any potential drivers and hindrances arising from the app design elements. Figure 1 illustrates the research model that guides this work. A set of confounding variables is also controlled for, given that demographics and anxiety have been found to influence the effectiveness of behavioral change [19].

## Methods

### Description of the mHealth App

**Figure 2:**
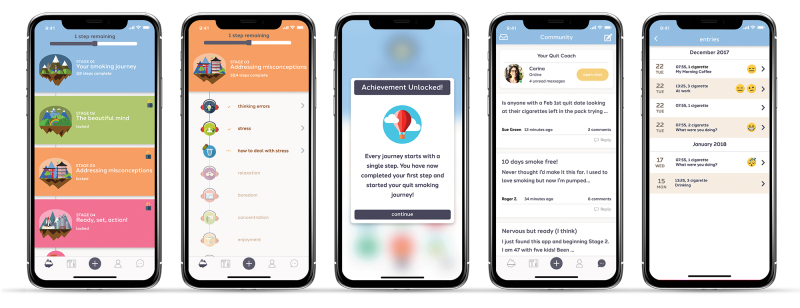
Screenshots of Quit Genius app.

We worked together with Quit Genius (QG), an mHealth intervention that delivers a digital CBT program to help people quit smoking. The digital CBT program is split into four stages consisting of 39 steps. The steps are made up of audio sessions, animated videos, reflective exercises and quizzes, which focus on identifying and altering the thoughts and behaviors that keep people smoking. Figure 2 illustrates QG’s gamification tactics; eg, challenge—ability balance, meaningful framing, personalization, fun and user-centric design. The protocol of this research received ethical approval by Imperial College London.

### Procedure

We designed and conducted an online survey, which was sent out to QG users via email in November and December 2017. To incentivize participation, we offered a free membership to the premium features of the QG app. This included access to additional relaxation techniques, post-quit date support and a social community to interact with other smokers. Furthermore, as part of the premium membership users have access to trained Quit Smoking Coaches who analyze their smoking patterns and share personalized tips to help app users with triggers that make them want to smoke and to help keep them motivated to quit smoking. Individuals received access to the premium features of the app after they completed the survey. Of 4144 survey recipients, 217 (5.24%) individuals responded to the survey. QG also provided the objective program progress data of respondents to the survey, following users’ consent. For each respondent, we matched the survey response data with program progress data (% completion rate of the app’s various steps as part of the quit smoking journey). Responses with excessive missing values were omitted, which resulted in a dataset of 190 completed responses.

### Online Survey

Whenever possible we employed multi-item scales from published work. Specifically, to capture hedonic well-being, we adapted three items from Ryff’s seminal work on psychological well-being [21] and Guevarra and Howell’s published hedonic well-being scale [22]. To capture psychological empowerment, we adapted four items from Dahl et al [25] and Park et al [26] published psychological empowerment and enablement scales, respectively. We measured inspiration and anxiety by adapting two items from Park et al [26] and Eisingerich et al [27] published work, respectively. To avoid respondents’ fatigue and manage response rate and quality, we kept the online survey as short as possible. Table 1 shows the detailed measurement items employed in the survey. The reliability of two item-measures was tested by assessing Pearson correlation coefficients; while the reliability of measures with more than two items were tested by examining the measures’ Cronbach’s alpha (Table 1).

**Table 1:** Measurement items and reliabilities.

Constructs and items *(1=not at all, 7=very much)*		Reliabilities
**Hedonic well-being**		*α=*.88
	Quit Genius contributes to my happiness today.		
	Quit Genius has increased my overall life satisfaction.		
	Quit Genius has helped me to be more productive at work.		
**Psychological empowerment**		*α=*.90
	Quit Genius has helped me think about smoking in a different way.		
	Quit Genius has helped me change my behavior.		
	Quit Genius has helped me increase my willpower to quit.		
	Quit Genius has helped me realize that mobile apps can improve my health.		
**Inspiration**		*r=*.82
	Quit Genius motivates me to be the person I want to be.		
	Quit Genius inspires me to live a healthier life.		
**Anxiety**		*r=*.50
	I am worried that Quit Genius won’t work for me.		
	I am afraid that Quit Genius could be a waste of my time and effort.		

### Statistical Analysis

We used the SPSS 24.0 software package for regression analyses. Binary logistic regression analyses were conducted to estimate the probability of being smoke-free after using the QG mHealth app. Furthermore, we explored the reduction in number of cigarettes smoked by regressing this variable on both program completion and the app gamification design elements, after having used a log transformation. Additionally, we set out to test to what extent progressing with the QG program equipped app users with a sense of well-being, empowerment and inspiration, thereby leading to these behavioral changes; ie, (1) success rate of being smoke-free and (2) number of cigarette reductions per day. Our first step was to examine the characteristics of the sample. Furthermore, we investigated QG’s gamification design and whether it provided three critical psychological benefits, namely empowering its users, enhancing their well-being, and inspiring them. This effect was compared between users who paid for the app and those users who used the free version of the app. The paid version of the app offers additional and personalized information. In addition, success rates of being smoke-free and reduction in cigarettes smoked per day were examined, respectively, by considering the ability of the app to empower users, boost their well-being, and inspire them, together with control variables including user anxiety, age, gender, and employment status.

## Results

### Characteristics of the Sample

**Figure 3:**
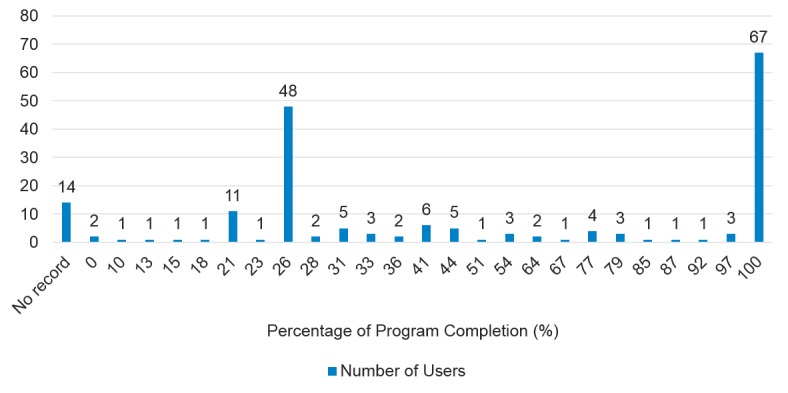
Cognitive behavioral therapy program completion at the time of the survey.

Progress of the program was examined by obtaining the objective usage data from QG. Specifically, for each respondent, the exact percentage of completion of the digital CBT program was obtained in relation to the number of steps of the program they had completed. The QG app has a total of 39 steps. Thus, if respondents completed all 39 steps at the time of the survey, they were assigned a score of 100% for completion. Figure 3 shows the number of users for each program progress at the time of survey. The largest group (67 of 190 respondents, 35.3%) of program completion were users who had completed the full program (100%). Of note, 26% of program completion is the second largest group (48 of 190 respondents, 25.3%) as this marks the end of the app’s free trial. A small group of respondents (14 of 190 respondents, .1%) had no record for program completion because they used different email addresses when they signed up for the survey and when they signed-up for the app.

The descriptive statistics for the final sample are reported in Table 2. In brief, respondents were on average 36 years old. Of the respondents, 52.6% (100/190) were female and 67.9% (129/190) were currently employed. On average, respondents had completed 60% of the digital CBT program in the QG app. Respondents had smoked an average of 15 cigarettes a day and had been smoking for an average of 17 years before using the QG app. Most of the respondents had tried to quit smoking before using the QG app (142/190, 74.7% tried to quit smoking up to 10 times; 19/190, 10.0% tried to quit smoking more than 10 times).

After using QG, 36.3% (69/190) of respondents noted to have quit smoking successfully. As for those remaining smoking after using the app (N=121), the number of cigarette reduced significantly by 59.6% (*M*_before_ 16.15, SD 7.89 vs *M*_after_ 9.63, SD 6.28; *t*_120_=10.97, *P*<.001). Correlation results of all noted variables are shown in Table 3.

### Relationship Between Program Completion and Psychological Benefits Offered by the App

As a first step, we regressed three potential psychological benefits offered by the app (empowerment, well-being, and inspiration) on program completion. The results (N=176) showed that the percentage of program completion of the app both significantly predicted enhanced user empowerment (*F*_1, 174_=13.87, beta=.27, *t*=3.72, *P*<.001) and user inspiration (*F*_1, 174_=5.91, beta=.18, *t*=2.43, *P*=.02). Users’ well-being afforded by the app was marginally associated with the percentage of program completion (*F*_1, 174_=3.60, beta=.14, *t*=1.90, *P*=.06).

### Relationship Between Users of the Paid vs Free Version of App and Psychological Benefits Offered by the App

As a second step, a set of *t*-tests was performed, which revealed that there was a significant difference between users of the paid vs the free version of the app in all three psychological outcomes afforded by the app. This included well-being (*M*_paid_ 4.26, SD 1.44 vs *M*_free_ 3.73, SD 1.59; *t*_174_=2.26, *P*=.03), empowerment (*M*_paid_ 5.55, SD 1.23 vs *M*_free_ 4.78, SD 1.44; *t*_174_=3.74, *P*<.001), and inspiration (*M*_paid_ 5.33, SD 1.43 vs *M*_free_ 4.83, SD 1.49; *t*_174_=2.22, *P*=.03). Thus, the results suggested that users of the paid version were more likely to receive the psychological benefits offered by the app, compared to users of the free version of the app.

### Successfully Quitting Smoking After Using the App

A binary logistic regression was performed to estimate whether the probability of being smoke-free after using the app (1=remaining a smoker, 2=being smoke-free) was associated with the different app benefits. Specifically, the results (see Table 4) demonstrated that both well-being (*P*=.01) and inspiration (*P*=.05) made a significant contribution to predicting the likelihood of quitting smoking.

When hedonic well-being was strengthened by one unit (1-point scale out of 7-point scales) the odds ratio was 1.72 times as large, and therefore respondents whose well-being was enhanced were 1.72 times *more* likely to quit smoking successfully. Interestingly, when inspiration was raised by one unit, the odds ratio was .66 times as small and thus a respondent who felt inspired by the app was 1.51 times more likely to remain a smoker (1/.66).

**Table 2:** Survey respondents: descriptive statistics. Likert scale anchors for users’ well-being, empowerment, inspiration, anxiety was from *1=not at all to 7=very much*.

Description	Mean (SD)
(1) Percentage of program completion	60.31(35.34)
(2) How many cigarettes did you smoke a day?	15.44 (8.47)
(3) How many years had you been smoking for?	16.92 (10.55)
(4) Enhancement of user well-being	4.13 (1.56)
(5) Empowerment of users	5.28 (1.38)
(6) Inspiration of users	5.18 (1.49)
(7) User anxiety	2.77 (1.38)
(8) Age	36.69 (10.23)
(9) Gender (1=*male*, 46%; 2=*female*, 53%; 3=*other*, 1%)	1.55 (.52)
(10) Are you currently in paid work? (1 =*no*, 32%; 2=*yes*, 68%)	1.68 (.47)
(11) Have you tried to quit in the past? (1=*no*, 15%; 2=*yes*, 85%)	1.85 (.36)
(12) Are you currently smoke-free? (1=*no*, 64%; 2=*yes*, 36%)	1.36 (.48)

**Table 3:** Correlation table.

Variable	1	2	3	4	5	6	7	8	9	10	11	12
(1) Program completion	1.00											
(2) How many cigarettes did you smoke a day?	-.04	1.00										
(3) How many years had you been smoking for?	.12	.33^a^	1.00									
(4) Have you tried to quit in the past? (1=*no*, 2=*yes*)	.02	.06	.03	1.00								
(5) Are you currently smoke-free? (1=*no*, 2=*yes*)	.11	-.11	-.08	-.06	1.00							
(6) Enhancement of user well-being	.14	.11	.07	-.09	.38^a^	1.00						
(7) Empowerment of users	.27^a^	.09	.09	-.02	.31^a^	.75^a^	1.00					
(8) Inspiration of users	.18^b^	.04	.07	-.07	.17^b^	.66^a^	.79^a^	1.00				
(9) User anxiety	-.12	-.07	-.03	.11	-.28^a^	-.52^a^	-.46^a^	-.30^a^	1.00			
(10) Age	.15^b^	.26^a^	.76^a^	.07	-.01	.03	.08	.05	.06	1.00		
(11) Gender (1=*male*, 2=*female*, 3=*other*)	.08	.17^b^	.16^b^	.01	-.09	-.08	.04	-.02	.06	.22^a^	1.00	
(12) In paid work? (1=*no*, 2=*yes*)	-.04	-.08	-.09	-.03	.11	-.07	-.004	-.04	.04	-.01	-.02	1.00

^a^Correlation is significant at the .01 level (2-tailed).

^b^Correlation is significant at the .05 level (2-tailed).

**Table 4:** Quitting smoking successfully after using the app (*χ*^2^_7_=40.4, N=190). App benefits and anxiety scored from 1 for *not at all* to 7 for *very much*. Gender scored 1 for *male*, 2 for *female*, 3 for *other*. In-paid work scored 1 for *no*, 2 for *yes*. Importantly, as shown in Table 5, a 1-unit increase on a 7-point Likert scale in the app’s ability to *empower* smokers in their daily lives led to a *greater reduction* in number of *cigarettes smoked* by 53% (beta=.53, *t*=3.26, *P*=.001). Moreover, the results showed that a 1 unit increase on a 7-point Likert scale in the app’s ability to *inspire* smokers led to a *weaker reduction* in the extent of cigarette reduction by 37% (beta=-.37, *t*=-2.58, *P*=.01).

Predicator		β (SE)		OR^a^	
**App benefits**					
	Well-being		.54 (.19)		1.72^b^	
	Empowerment		.41 (.25)		1.50	
	Inspiration		-.41 (.21)		.66^c^	
**Control**					
	Anxiety		-.16 (.15)		.85	
	Age		.00 (.02)		1.00	
	Gender		-.31 (.35)		.73	
	In-paid work		.73 (.38)		2.07	
Constant		-3.23 (1.39)		.04^d^	

^a^OR: odds ratio.

^b^*P*<.01.

^c^*P*=.05.

^d^*P*<.05.

**Table 5:** Reduction in number of cigarettes smoked for remaining smokers (R^2^=.21, F_7, 113_=4.28, *P*<.001).

Predictor		β (SE)	*t*
**App benefits**			
	Well-being	.18 (.04)	1.36
	Empowerment	.53 (.05)	3.26^a^
	Inspiration	-.37 (.04)	-2.58^b^
**Control**			
	Anxiety	.05 (.03)	.52
	Age	.06 (.01)	.69
	Gender	.07 (.08)	.77
	In-paid work	-.08 (.08)	-.90
Constant		.05 (.28)	.17

^a^*P*<.001.

^b^*P*<.05.

### Remaining a Smoker after Using the App

For those who remained smokers (N=121), any changes in the number of cigarettes they smoked was explored. Firstly, we normalized the number of cigarette reduction by using log transformation and then regressed this outcome variable on both program completion and app elements. As shown in Table 5, a significant regression equation was observed (*F*_7, 113_=4.28, *P*<.001) with an R^2^of .21.

### Mediation Analyses

A set of mediation tests were conducted to examine: (1) program completion, three psychological benefits, number of cigarette reduction; and (2) program completion, three psychological benefits, probability of being smoke free by using syntax *binary mediation* in Stata 14.2 which allows either continuous or dichotomous outcome variables together with multiple mediators. Table 6 shows the detailed mediation test results.

**Table 6:** Mediation test results. Model 1 tests the mediational role of well-being, empowerment, and inspiration as mediating the relationship between program completion (as independent variable) and number of cigarette reduction per day (as dependent variable). In addition to the indirect effects, a direct effect of program completion on cigarette reduction was reported. Model 2 tests the mediational role of well-being, empowerment, and inspiration as mediating the relationship between program completion (as independent variable) and the probability of being smoke-free (as dependent variable). In addition to the indirect effects, a direct effect of program completion on the probability of being smoke-free was reported.

Mediator variable and model				*R* ^2^	*F* (*df*)	*χ*^2^(*df*)	β (SE)	*t*	Z	95% CI
**(1) Cigarette reduction**										
	**Mediator variable model**									
		**Predicating well-being**								
			Program completion	N/A^a^	N/A	N/A	.00 (.01)	1.32	N/A	N/A
		**Predicating empowerment**								
			Program completion	N/A	N/A	N/A	.01 (.01)	2.88^b^	N/A	N/A
		**Predicating inspiration**								
			Program completion	N/A	N/A	N/A	.01 (.01)	1.34	N/A	N/A
	**Dependent variable model**									
		**Predicating cigarette reduction**								
			Program completion	.00	.03 (1, 113)	N/A	.00 (.00)	.17	N/A	(-.00 to .00)
	**Dependent variable model**									
		**Predicating cigarette reduction**								
			Program completion	.15	6.18 (4, 110)^e^	N/A	-.00 (.00)	-1.11	N/A	(-.00 to .00)
			Well-being	N/A	N/A	N/A	.05 (.04)	1.21	N/A	(-.03 to .13)
			Empowerment	N/A	N/A	N/A	.18 (.05)	3.33^b^	N/A	(.07 to .29)
			Inspiration	N/A	N/A	N/A	-.11 (.04)	-2.62^c^	N/A	(-.20 to -.03)
**(2) Probability of being smoke-free**										
	**Mediator variable model**									
		**Predicating well-being**								
			Program completion	N/A	N/A	N/A	.01 (.00)	1.90^d^	N/A	N/A
		**Predicating empowerment**								
			Program completion	N/A	N/A	N/A	.01 (.00)	3.72^e^	N/A	N/A
	**Mediator variable model**									
		**Predicating inspiration**								
			Program completion	N/A	N/A	N/A	.01 (.00)	2.43^c^	N/A	N/A
	**Dependent variable model**									
		**Predicating being smoke-free**								
			Program completion	N/A	N/A	2.10 (1)	.01 (.00)	N/A	1.44	(-.00 to .02)
	**Dependent variable model**									
		**Predicating being smoke-free**								
			Program completion	N/A	N/A	28.93 (4)	.00 (.01)	N/A	.59	(-.01 to .01)
			Well-being	N/A	N/A	N/A	.54 (.18)	N/A	2.93^b^	(.18 to .90)
			Empowerment	N/A	N/A	N/A	.47 (.25)	N/A	1.87^c^	(-.02 to .96)
			Inspiration	N/A	N/A	N/A	-.48 (.21)	N/A	-2.28^c^	(-.89 to -.07)

^a^N/A: not applicable.

^b^*P*<.01.

^c^*P*<.05.

^d^*P*<.10.

^e^*P*<.001.

## Discussion

### Key Findings

**Figure 4:**
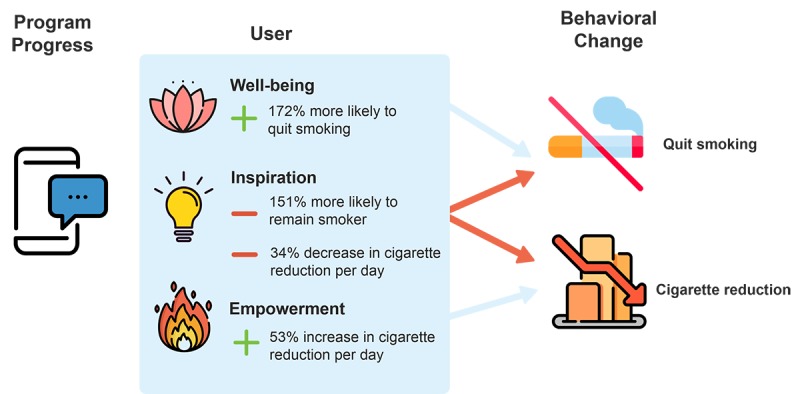
Main findings of this research.

Figure 4 summarizes the key findings of this study. Thus, in the context of an mHealth app that helps users quit smoking, we show that 36% of QG users were self-reported to have successfully quit smoking, and of those who remained smokers, the number of cigarettes was reduced significantly by 59.6%. In addition, we found that progress in QG’s mHealth program was associated with enhanced user hedonic well-being (contributing to their overall life satisfaction, happiness, and helping them to be more productive at work).

Furthermore, progress in QG’s mHealth program was associated with enhanced user empowerment (helping them think about smoking in a different way, increasing their willpower to quit, and changing their behaviors, and realizing that mobile apps can improve their health). Moreover, the results showed that progress in QG’s mHealth program was associated with enhanced user inspiration (motivating them to be the person they want to be, inspiring them to live a healthier life).

A key finding of the current research is that the success rate of users quitting smoking was significantly enhanced through gamification designs that boosted their hedonic well-being, whereas user inspiration by the gamification designs was negatively associated with quitting smoking successfully. Additionally, the results demonstrated that mHealth gamification design that empowered app users was associated with a greater reduction in cigarettes smoked per day, whereas, surprisingly, inspiration was negatively associated with a reduction in cigarettes smoked per day.

### Extending Extant Knowledge

The findings of this study offer relevant and potentially provocative insights into the effective design for digital mHealth apps [17,18]. The findings related to which elements of app design are more conducive to smoking cessation are intriguing and extend current knowledge about the design of evidence-based smoking cessation mHealth apps in important ways [18]. Contrary to common intuition, the inspiring element of the app was *negatively* associated with both the probability of successfully quitting smoking as well as the reduction in cigarettes smoked per day.

While inspiration can motivate and encourage individuals to keep going [23,24], it can also reduce an individual’s sense of self-responsibility at times. As prior research notes [26], inspiration is not the same as positive affect. People who enter an inspired state (by thinking of a moment when they were inspired) reported lower levels of volitional control, controllability, and self-responsibility for their inspiration. This ties in with the notion that smoking is not simply a bad habit—it is an addiction which is often derived from the fact that smokers desire to escape the reality [28-30]. When the inspiring elements of an mHealth app come into play, such a psychological state can be taxing to the extent that individuals are potentially encouraged to smoke to comfort themselves.

In addition to the app design elements that were shown to positively or negatively affect smoking cessation, progress in the QG’s gamified program journey was identified as a significant means to enhance users’ hedonic well-being, psychological empowerment, and inspiration. These finding shed light on the impact that completing the program has on behavioral change and underscores the role played by empowerment, inspiration and well-being afforded by the gamification design elements of the app.

The results of this study further extend prior gamification design research of mobile apps for smoking cessation [18,31-33], showing how the design of mHealth apps is highly relevant for exploring new methods and facilitating new ways of encouraging a healthy lifestyle. In line with the literature [29,34-36], the success or failure of digital services such as a mobile phone app that tries to encourage a healthier lifestyle is highly dependent on the app’s efficacy in achieving behavioral change. Based on research exploring game-based health interventions [18,19], as well as theories and practice of designing for change [19], our findings suggest that in the context of smoking cessation, psychological empowerment through the gamified design of an mHealth app significantly predicts users’ ability to quit smoking successfully.

To date, most of prior work has focused on the usability and feasibility of mobile apps as mHealth interventions for self-guided care in smoking cessation [30-33]. Our focus differs from this approach, as we investigate a more psychological side of effectiveness in addition to behavioral therapy. Building on work which highlights the important role played by psychological benefits to the self of individuals [34-39], our goal was to explore and extend current work on various mHealth app design elements to maximize the effectiveness on behavioral outcomes [40-42].

### Limitations and Future Research

The current findings need to be viewed with the following limitations in mind. First, the self-report method of tracing back users’ previous smoking behavior (eg, how many cigarettes they smoked before and after using the app) could result in an estimation bias. We encourage additional work to investigate users’ actual behavioral change in the context of digital health across longitudinal studies using biological measures. Moreover, due to the lack of data we could not account for the time since participants had their last cigarette. Future research that also captures participants’ time intervals between smoking cigarettes and their last cigarette smoked (eg, hazard ratio model) can help shed additional light on the current findings.

Second, we note the potential of response bias, as the users who were more motivated and engaged with the app might be more likely to complete the survey. While the response rate of this research is in line with other work based on online surveys [43,44], we invite future research to strengthen confidence in the generalizability of the current results by replicating the study with a larger sample and a higher survey response rate.

Third, to achieve a parsimonious conceptual model and to keep the online survey short to manage response rate and quality, we did not include other measures that could inform our analyses of user-app engagement and efficacy. The negative association between inspiration elements of the app and success rate of quitting smoking, as well as reduction in cigarettes smoked per day is curious. One wonders about potential process mechanisms at play here. Why does inspiration hinder and not help in the context of smoking cessation? When, if at all, does inspiration help smokers quit? How does inspiration impact one’s willpower to quit smoking and their desire for relief? We invite future research to address these intriguing questions.

Fourth, we conducted this study in one context (smoking cessation) and future research that extends our work to other contexts (weight loss, reducing alcohol consumption, pornography, shopping, gambling addiction, as well as addiction to social media such as Instagram, Tinder, Facebook, and more) in which mobile health interventions could be used is richly deserving. Fifth, we invite future research to test the effectiveness of digital mHealth apps against current interventions (eg, nicotine replacement therapy) in smoking cessation, which might help drive culture change and openness to different mHealth solutions.

Finally, given the real potential of digital transformation of human life in the future [45] and individuals’ current willingness to exert significant energy (emotional, time, money, and more) online [46], we call for more research to study the effects of digital applications on human well-being. As day-to-day experiences and casual observation suggest, more and more people appear to get “hooked” on digital devices and their apps and cannot imagine their lives without these [47]. Developing apps that truly improve a person’s well-being is undoubtedly an ambitious goal (just as is spotting the rainbow unicorn in the start-up scene), and thus we encourage additional research to join this important effort. Let us create a future where digital solutions do not enslave humans but help them be more productive, increase overall life satisfaction, and help lead healthier, more fulfilling lives.

### Conclusions

The results of this research highlight that progress in QG’s mHealth program was associated with stronger user hedonic well-being, greater empowerment, and enhanced user inspiration. Furthermore, the results show that users’ ability to quit smoking successfully was significantly enhanced through QG’s gamified mHealth elements that strengthened their well-being. In contrast and curiously, inspiration offered by the QG app was negatively associated with quitting smoking successfully. Possibly, in the context of smoking cessation inspiration adds to the level of stress and anxiety of smokers rather than helping them in their efforts to quit. Thus, taken together the current findings offer critical implications to the effective design of mobile health solutions, such as digital apps geared towards improving users’ health. In the context of smoking cessation, we find there is real value in helping users think about smoking in a different way (empowerment) and increasing their overall life satisfaction (a sense of well-being).

## Abbreviations

CBT: cognitive behavioral therapy
mHealth: mobile health
QG: Quit Genius
